# Unsupervised Machine Learning for Identifying Challenging Behavior Profiles to Explore Cluster-Based Treatment Efficacy in Children With Autism Spectrum Disorder: Retrospective Data Analysis Study

**DOI:** 10.2196/27793

**Published:** 2021-06-02

**Authors:** Julie Gardner-Hoag, Marlena Novack, Chelsea Parlett-Pelleriti, Elizabeth Stevens, Dennis Dixon, Erik Linstead

**Affiliations:** 1 Schmid College of Science and Technology Chapman University Orange, CA United States; 2 Center for Autism and Related Disorders Woodland Hills, CA United States; 3 Fowler School of Engineering Chapman University Orange, CA United States

**Keywords:** autism spectrum disorder, challenging behaviors, unsupervised machine learning, subtypes, treatment response, autism, treatment, behavior, machine learning, impact, efficacy, disorder, engagement, retrospective

## Abstract

**Background:**

Challenging behaviors are prevalent among individuals with autism spectrum disorder; however, research exploring the impact of challenging behaviors on treatment response is lacking.

**Objective:**

The purpose of this study was to identify types of autism spectrum disorder based on engagement in different challenging behaviors and evaluate differences in treatment response between groups.

**Methods:**

Retrospective data on challenging behaviors and treatment progress for 854 children with autism spectrum disorder were analyzed. Participants were clustered based on 8 observed challenging behaviors using *k* means, and multiple linear regression was performed to test interactions between skill mastery and treatment hours, cluster assignment, and gender.

**Results:**

Seven clusters were identified, which demonstrated a single dominant challenging behavior. For some clusters, significant differences in treatment response were found. Specifically, a cluster characterized by low levels of stereotypy was found to have significantly higher levels of skill mastery than clusters characterized by self-injurious behavior and aggression (*P*<.003).

**Conclusions:**

These findings have implications on the treatment of individuals with autism spectrum disorder. Self-injurious behavior and aggression were prevalent among participants with the worst treatment response, thus interventions targeting these challenging behaviors may be worth prioritizing. Furthermore, the use of unsupervised machine learning models to identify types of autism spectrum disorder shows promise.

## Introduction

Autism spectrum disorder is a neurodevelopmental disorder characterized by deficits in social communication and social interaction, as well as the presence of restricted, repetitive patterns of behavior, interests, and activities [1]. With the exception of restricted, repetitive behaviors (eg, stereotypy, perseveration), challenging behaviors are not classified as a core symptom of autism spectrum disorder; however, these behaviors are prevalent among individuals with autism spectrum disorder. As many as 94% of children with autism spectrum disorder engage in some type of challenging behavior, often including stereotypy (eg, self-stimulatory or persistent repetitive motor or vocal behavior), aggression, tantrums, and self-injurious behavior [2,3]. Challenging behaviors may pose risk of injury to the individual or others and may inhibit learning opportunities and social interactions [4]. Furthermore, challenging behaviors may negatively impact family functioning and contribute to caregiver stress [5,6].

Various risk factors for engagement in challenging behaviors have been investigated in individuals with autism spectrum disorder. Symptom severity has been found to predict challenging behaviors, with greater symptom severity associated with engagement in higher numbers of challenging behaviors at stronger intensities [2,3]. Intellectual functioning has also been linked to challenging behaviors in individuals with autism spectrum disorder, with greater deficits in intellectual functioning predicting greater frequencies of stereotypy [7,8], aggression [8], and self-injurious behavior [8,9]. In addition, deficits in adaptive skills [10,11] and expressive language skills [11] have been associated with engagement in challenging behaviors in individuals with autism spectrum disorder, but studies [8-12] that investigated the relationship between gender and challenging behaviors found no significant differences in engagement in challenging behaviors between boys and girls with autism spectrum disorder.

Applied behavior analysis interventions, which involve the application of principles and procedures of learning and motivation to alter behavior [13,14], may be used to reduce challenging behaviors and increase appropriate behaviors in individuals with autism spectrum disorder. Specific challenging behaviors that are commonly addressed in treatment include stereotypy, noncompliance, and aggression [15]. Outcome studies for children with autism spectrum disorder have not often included challenging behaviors as an outcome measure [4,16]. Several group design studies [17-19] have found evidence to support the use of caregiver training to manage challenging behaviors. Furthermore, there is an abundance of single-individual research evaluating the effectiveness of behavioral interventions for challenging behaviors in individuals with autism spectrum disorder, and reviews of this research have found behavioral interventions, particularly those implementing pretreatment functional assessments, to be effective in reducing challenging behaviors [20-22].

Applied behavior analysis–based therapy is considered to be well-established for the treatment of autism spectrum disorder [23,24]. While ample research demonstrates the effectiveness of applied behavior analysis–based treatment [25,26] research also reveals variability in individual response to treatment [27,28]. Treatment-related variables including greater treatment intensity [27,29-32], longer treatment duration [30-32], and greater total intervention time [33,34] have been linked to superior treatment outcomes. Furthermore, many patient-related variables have been associated with greater treatment gains. These include younger age [29,32,34-38], lower autism spectrum disorder symptom severity [35,36,38,39], and greater intellectual functioning [27,36,38-45].

Research evaluating the impact of challenging behaviors on treatment response in individuals with autism spectrum disorder is limited. Eikeseth and colleagues [46] investigated whether challenging behaviors, among other intake measures, were associated with treatment outcomes for adaptive behavior and autism spectrum disorder symptoms in children with autism spectrum disorder; however, challenging behaviors were not found to be a predictor of treatment outcome. Conversely, Remington and colleagues [39] found that higher rates of challenging behaviors at intake were associated with superior response to treatment and suggested that their counterintuitive findings may possibly be attributed to the sensitivity of the measure used to assess challenging behaviors. Given the prevalence of challenging behaviors among individuals with autism spectrum disorder, additional research is needed to investigate the impact of these behaviors on treatment response.

To account for the heterogeneity observed across individuals with autism spectrum disorder, researchers have investigated types of autism spectrum disorder [47]. Preliminary research has found behavioral types of autism spectrum disorder to have differences in gene expression [48-50], developmental trajectory [51-54], and treatment response [55]. In a recent study, Stevens and colleagues [55] used an unsupervised machine learning model to identify behavioral types of autism spectrum disorder and evaluate differences in treatment response across types. Participants included 2400 children with autism spectrum disorder. Data from a comprehensive assessment of skill deficits and treatment progress data were analyzed. A total of 16 autism spectrum disorder groups were identified using a Gaussian mixture model. Using a linear regression model, relationships between treatment hours and skill mastery were found to be strong within groups, accounting for 64% to 75% of variance. These findings are a preliminary step toward advancing targeted treatments and improving outcomes for individuals with autism spectrum disorder based on type membership.

Autism spectrum disorder types may also be identified based upon profiles of challenging behavior. Stevens and colleagues [56] conducted an analysis of challenging behaviors in a large sample of children with autism spectrum disorder (n=2116). Using *k*-means clustering, 8 diverse profiles, in which a single dominant challenging behavior was apparent, were identified. Furthermore, gender differences were observed when cluster analyses were performed separately for male and female participants. While all of the male clusters were found to exhibit a single dominant challenging behavior, 2 of the female clusters indicated equal engagement in 2 dominant challenging behaviors. These findings suggest that gender may play a role in the presentation of challenging behaviors in individuals with autism spectrum disorder. Further investigations into autism spectrum disorder types based on challenging behaviors are warranted.

The study of challenging behaviors across types of autism spectrum disorder may help explain some of the variation observed in treatment outcomes across individuals with autism spectrum disorder and may advance efforts to develop targeted treatments to maximize outcomes. Preliminary evidence indicates there are autism spectrum disorder types based on challenging behaviors; however, little is known about how challenging behaviors impact treatment response. The purpose of this study was to identify types of autism spectrum disorder based on engagement in different challenging behaviors and evaluate differences in treatment response between groups and across gender.

## Methods

### Data Set

Deidentified retrospective treatment data for a large sample of children with autism spectrum disorder were used in this study. Data on the frequency of challenging behaviors and treatment progress were obtained from the Skills system software (Skills Global LLC [57]). Skills includes a thorough assessment of skill deficits with demonstrated reliability [58] and validity [59], a comprehensive curriculum to build individualized treatment plans, and tracking capabilities for challenging behaviors and ongoing treatment progress. In addition to Skills data, operational data on treatment hours were used in this study.

Participants included children with autism spectrum disorder who were receiving applied behavior analysis treatment from a community-based provider. A total of 2116 clinical records were reviewed based on the following inclusion criteria: (1) were between the ages 18 months and 12 years old; (2) had a diagnosis of autism spectrum disorder, autistic disorder, pervasive developmental disorder–not otherwise specified, or Asperger syndrome by an independent licensed clinician (eg, psychologist, pediatrician, etc); (3) received at least 20 hours of treatment per month; and (4) had at least 1 month of continuous services; (5) demonstrated repeated instances of challenging behavior as documented in their treatment history; and (6) had available treatment response data over the course of treatment. Parameters with respect to age were set based on the age range predominately represented in the data set to avoid potential outliers that may have affected the cluster analysis. Likewise, parameters regarding treatment intensity and duration were established so that each participant had adequate treatment response data to include in the analysis. After applying these criteria, a sample of 854 participants were included. Of the participants, 706 were male and 148 were female. The average age of participants was 7.59 (SD 2.17) years old, ranging from 2.74 years to 12 years. Participants resided in the states of Arizona, California, Colorado, Illinois, Louisiana, New York, Texas, and Virginia. The data used for this study were collected during a 36-month period (January 1, 2014 through December 31, 2016).

### Measures

Data on engagement in challenging behaviors were used to identify potential clusters. While the classification of challenging behaviors is subjective in nature, there is agreement among the literature regarding operational definitions for common topographies of challenging behaviors exhibited by individuals with autism spectrum disorder [4]. While this may not be exhaustive, data were examined for the following topographies of challenging behaviors: aggression (eg, hitting, kicking), disruption (eg, interrupting, yelling), elopement (eg, wandering, bolting), noncompliance (eg, defiant behavior, refusing), obsessive behavior (eg, repeatedly talking about the same topic, preservation), self-injurious behavior (eg, head banging, hand biting), stereotypy (eg, hand flapping, toe walking, vocal stereotypy), and tantrums (eg, crying, falling). Skills is implemented as a relational database, which allows behavior interventionists to record observations in real time during a therapy session using an iPad and the corresponding Skills app. In the case of challenging behaviors, when such a behavior is observed, the therapist marks the type of behavior and provides a textual description of its context. This information is then timestamped and then stored in the underlying relational database. Aggregation of challenging behavior data for each patient can then be easily achieved using a simple database query (SQL format). An extra validation step was taken to compare identified challenging behaviors to the textual description provided by the behavior interventionist to ensure no challenging behavior observations were misidentified.

Data on mastered learning objectives were used to evaluate treatment response. Mastery criteria for learning objectives were determined by the patient’s clinician and individualized to the patient. Typically, mastery was defined as 80% accuracy or greater for a minimum of 2 treatment sessions across 2 days.

### Treatment

Participants received individualized applied behavior analysis–based treatment. Treatment comprehensively targeted deficits across developmental domains, including language, social, adaptive, cognitive, executive function, academic, play, and motor skills. Services were provided in the participant’s home, clinic, school, community, or a variety of settings. Treatment was provided according to the Center for Autism and Related Disorders model [60].

Participants’ treatment programs addressed skill acquisition and targeted the reduction of challenging behaviors. Interventions for challenging behaviors varied based on the target behavior’s topography and function (determined using functional assessment). Possible interventions implemented by a participant’s clinician included: antecedent-based interventions (ie, manipulations to the environment to reduce the target behavior) such as noncontingent reinforcement, demand fading, task modification, and choice; replacement behavior interventions including functional communication training, differential reinforcement of alternative behavior, and differential reinforcement of incompatible behavior; and consequence-based interventions (ie, manipulations to the events following the target behavior to reduce the likelihood of its reoccurrence) such as extinction, differential reinforcement of other behavior, differential reinforcement of low rate behavior, and response interruption and redirection.

### Data Analysis

#### Clustering

This analysis expanded on the work of Stevens and colleagues [56] to explore differences in treatment response across identified challenging behavior clusters in individuals with autism spectrum disorder. Patients were clustered based on relative frequency of 8 challenging behaviors (aggression, disruption, elopement, noncompliance, obsessive behavior, self-injurious behavior, stereotypy, and tantrums) using a *k*-means machine learning algorithm. This was achieved by creating an 8-dimensional feature vector for each patient. Relative frequency was calculated by finding the proportion of all challenging behaviors for each of the 8 categories for each patient. Duration and severity of the challenging behaviors were not taken into consideration for this value. Each vector element corresponded to the relative frequency of a specific challenging behavior observed for that patient. The 8-dimensional vectors were fed directly to the clustering algorithm without the use of feature selection because the dimensionality of the data was relatively small, and it was important to preserve each of the challenging behaviors in the final cluster model. Once clusters were identified using the *k*-means algorithm, multiple linear regression was performed to evaluate interactions between cluster assignment, treatment response, and gender.

The goal of clustering is to find latent groups, or clusters, in data. Patients within the same cluster will have more similar challenging behaviors profiles than patients in different clusters [61]. The *k-*means methods was selected for clustering because it is computationally efficient, easily implemented, and is a widely used prototype-based clustering algorithm, wherein each cluster is represented by a prototype. This prototype can either be the centroid of data points with similar continuous features or the medoid in the case of categorical features. This data set involved continuous features; therefore, each cluster had a centroid.

The *k*-means algorithm was implemented with 5 steps. (1) The best value of *k* (ie, the number of clusters) was identified by incrementally testing values between 2 and 20. (2) For each of these values, the algorithm picked *k* sample points from the data at random, which are the initial centroids (*c*_1_, *c*_2_, ..., *c*_k_). (3) Each 8-dimensional data point *d*_i_ was assigned to the nearest centroid *c*_k_ using Euclidean distance to measure the distance between the point and the centroid. (4) The algorithm recalculated the centroids by taking the mean value (for each behavior) from all the data points currently in the cluster. (5) The algorithm repeated steps 3 and 4 until the cluster assignments did not change or a maximum number of iterations was reached.

To find the distance between the data points and the centroids in the data set, squared Euclidean distance was used. Similarity between data points is defined as the opposite of distance. A commonly used metric for finding the distance between data points *x* and *y* in *m*-dimensional space is the squared Euclidean distance.

Once similarities are measured, clustering becomes an optimization problem. An iterative approach was used to minimize the within-cluster sum of squared errors or cluster inertia. Once these errors were calculated, a graph of the errors were examined using the elbow method to find the best value for *k*. The elbow method involves examining the plot (ie, the arm) to determine the point at which diminishing returns are observed (ie, the elbow). As *k* increases, the sum of squared errors gets smaller. When *k* is equal to the number of points in the data set, the sum of squared errors is 0 and every point is its own cluster. Choosing *k* to correspond to the elbow in the graph thus provides an effective measure by which to prevent overfitting. The chosen *k* indicates the optimal number of clusters that are both cohesive and separate.

#### Linear Regression

A multiple linear regression model was used to evaluate the relationships between the target variable (skill mastery) and explanatory variables (treatment intensity, cluster assignment, and gender).

In univariate linear regression, the relationship between a single explanatory variable *x* and a response or target variable *y* is modeled. The equation used for linear models with only 1 predictor variable is defined as y_i_=β_0_+ β_1_x_i_+ε_i_, where the weight β_0_ represents the *y*-axis intercept and β_1_ is the coefficient of the explanatory variable. In simple linear regression, the goal is to find the weights of the equation to explain the relationship between the explanatory variable and the target variable. From this, the responses of new data points that were not part of the observed data may be predicted and coefficients of the model may be interpreted. The simple linear regression equation may be generalized to produce an equation for multiple linear regression that involves multiple explanatory variables.

Linear regression works by taking the explanatory variables and the response variable, and fitting a straight hyperplane to the data that minimizes the distance between an observed point and the fitted model. The explanatory variables were treatment intensity, cluster assignment, and gender, and the response variable was skill mastery.

An efficient way to quantitatively measure a model's performance is the mean squared error, which measures the average squared error between the model’s prediction and the actual values. Mean squared error may be used to compare different regression models with the same outcome.

*R*^2^ (another measure of model fit) is bounded between 0 and 1, with 1 indicating a perfect relationship between *x* and *y* and mean squared error is equal to 0. *R* allows for the specification of interaction terms in regression formulas. An interaction occurs when the product of 2 predictor variables is also a significant predictor [62]. In this study, there were 3 explanatory variables (treatment intensity, cluster assignment, and gender), and all interactions were included in the model.

#### Tukey Posthoc

Results from the regression model indicated that there was a significant difference between treatment hours, cluster assignment, and the interaction between cluster assignment and gender. Posthoc analysis was conducted to determine which pairs of clusters significantly differed. The Tukey honestly significant difference method was used to correct for multiple comparisons.

The Tukey posthoc test assesses all the pairwise comparisons using the Tukey honestly significant difference formula

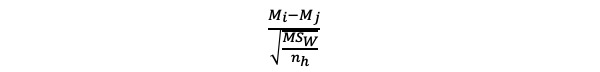



where *M_i_* – *M_j_* is the difference between the pairs of means, *MS_w_* is the mean square within, and *n* is the number of clusters.

## Results

### Clustering

**Figure 1:**
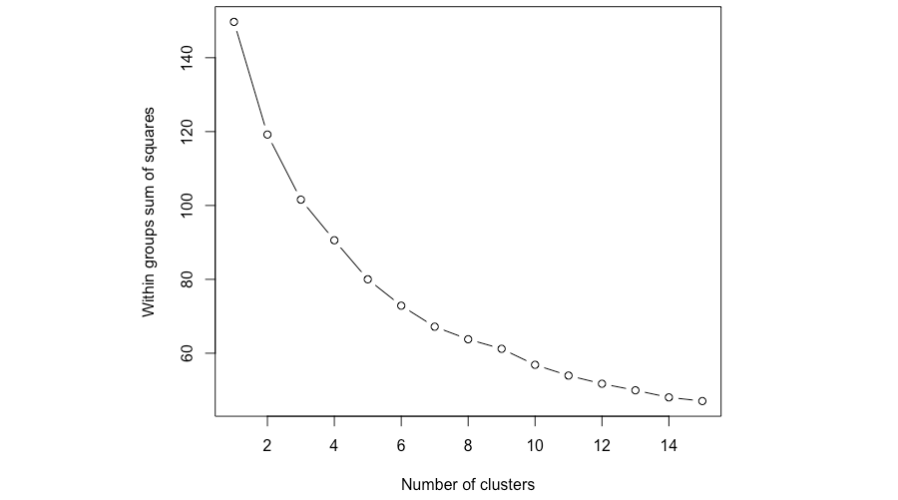
Within-cluster sum of squared errors for all patients, both male and female. The elbow method indicates that the best value of *k* is 7, meaning there are 7 clusters.

Figure 1 shows within-group sum of squared errors for patients. The optimal value of *k* (the number of distinct challenging behavior profiles) was found to be 7, confirmed both by the elbow method and by silhouette score (the highest indicates most cohesive and separate). Each cluster corresponds to a phenotype and can be quantitatively represented with its centroid (the mean relative frequency for each of the 8 challenging behaviors for patients in that cluster). The dimensionality of each centroid is identical to the input feature space, which is preserved during the clustering process.

Seven phenotypes of autism spectrum disorder, most of which demonstrated 1 dominant challenging behavior, were identified based on average frequency (centroid) of 8 challenging behaviors (ie, aggression, disruption, elopement, noncompliance, obsessive behavior, self-injurious behavior, stereotypy, and tantrums) calculated for each cluster (Table 1). It is important to reiterate that the machine learning process is unsupervised. The phenotypes are identified by the algorithm without the need for human labels, which are required for supervised learning (classification).

**Table 1:** Breakdown of identified clusters.

Cluster	Dominant challenging behavior	Boys, n	Girls, n	All, n
1	Tantrums	60	14	74
2	Self-injurious behavior	79	8	87
3	Elopement	78	18	96
4	Stereotypy (low rate)	170	37	207
5	Noncompliance	87	26	113
6	Aggression	113	26	139
7	Stereotypy (high rate)	119	19	138

**Figure 2:**
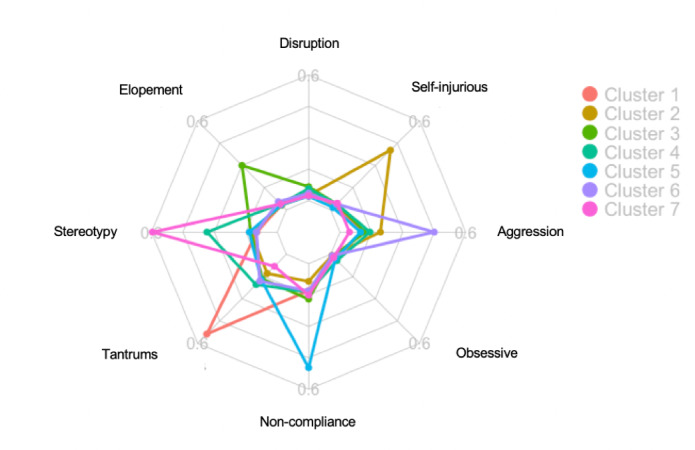
Radar graphs depicting engagement in challenging behaviors across clusters.

**Figure 3:**
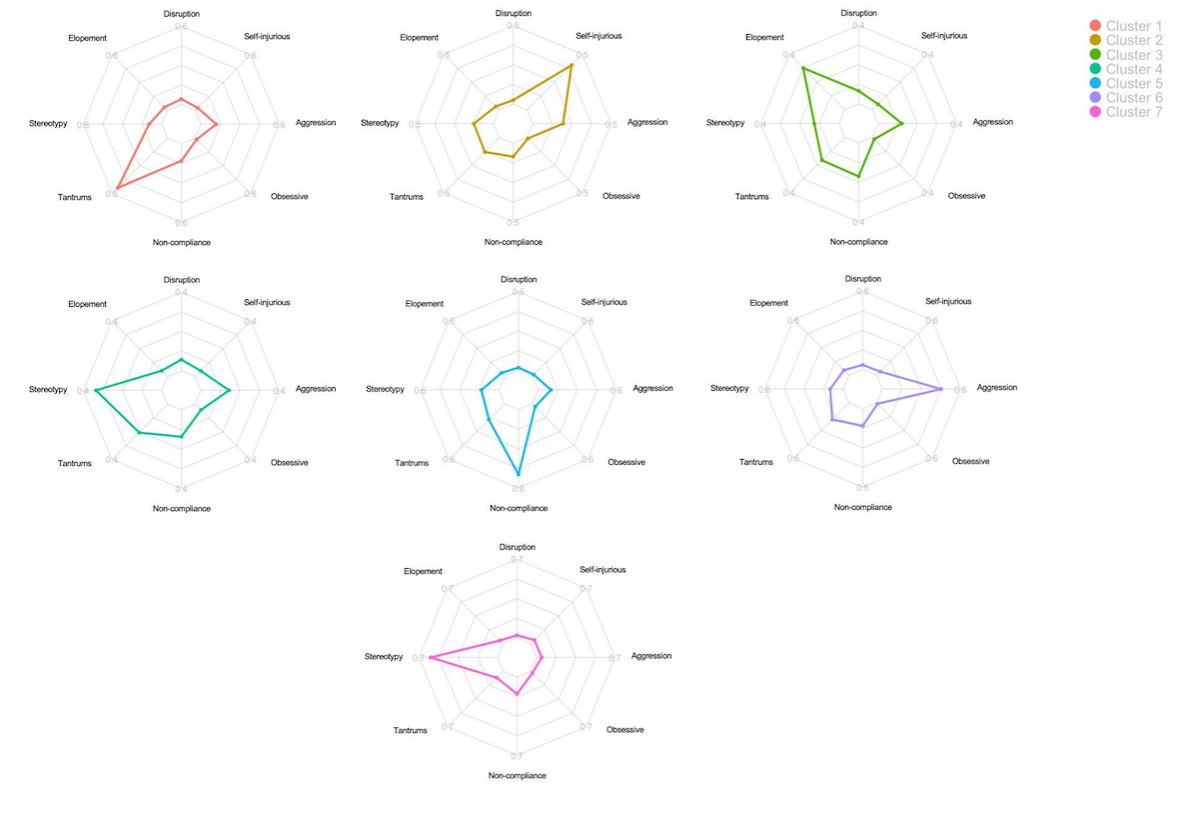
Radar graphs showing the dominant challenging behavior for each cluster. Note that the maximum varies between the clusters, particularly Cluster 4 and Cluster 7, in which patients demonstrate the same dominant challenging behavior.

The radar graphs shown in Figure 2 and Figure 3 provide visual representations of each phenotype’s engagement in the 8 challenging behaviors. The radar charts in Figure 3 were scaled from 0 to the average frequency of the dominant challenging behavior. For example, Cluster 1 was scaled from 0 to 0.6, to which tantrums extend. Cluster 4 was scaled from 0 to 0.4, to which stereotypy extends. It is worth noting that Cluster 4 and Cluster 7 both have stereotypy as their dominant challenging behavior, but their frequencies were different. Cluster 4 was found to engage in stereotypy at a lower rate than Cluster 7.

### Linear Regression

The *R*^2^ value was found to be 0.67. The value for *R*^2^ is the fraction of the variance of exemplar mastery that is explained by the model. Thus, the model explained 67% of the variance of exemplar mastery. The model was significantly predictive of mastery (*F*_27,826_=61.05, *P*<.001).

**Figure 4:**
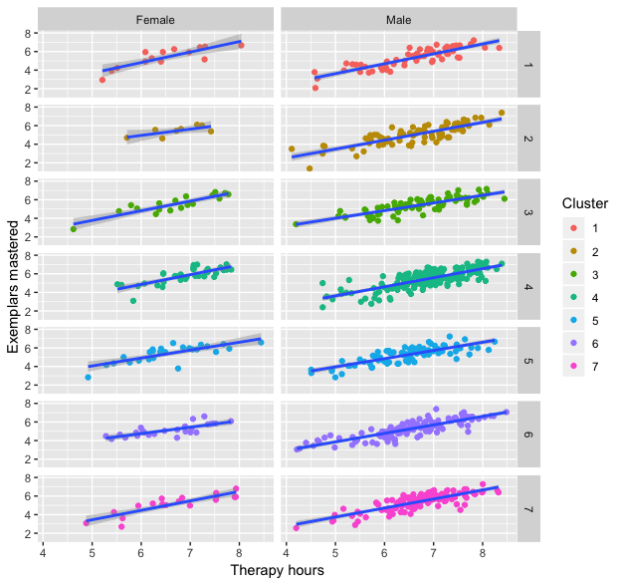
The line of best fit for each gender and cluster.

Figure 4 shows the regression lines for male and female patients in each of the different clusters. The mean squared error for each cluster is shown in Table 2.

**Table 2:** Mean squared error comparison across clusters.

Cluster	Dominant challenging behavior	Mean squared error
1	Tantrums	0.30
2	Self-injurious behavior	0.34
3	Elopement	0.23
4	Stereotypy (low rate)	0.37
5	Noncompliance	0.30
6	Aggression	0.24
7	Stereotypy (high rate)	0.29

**Figure 5:**
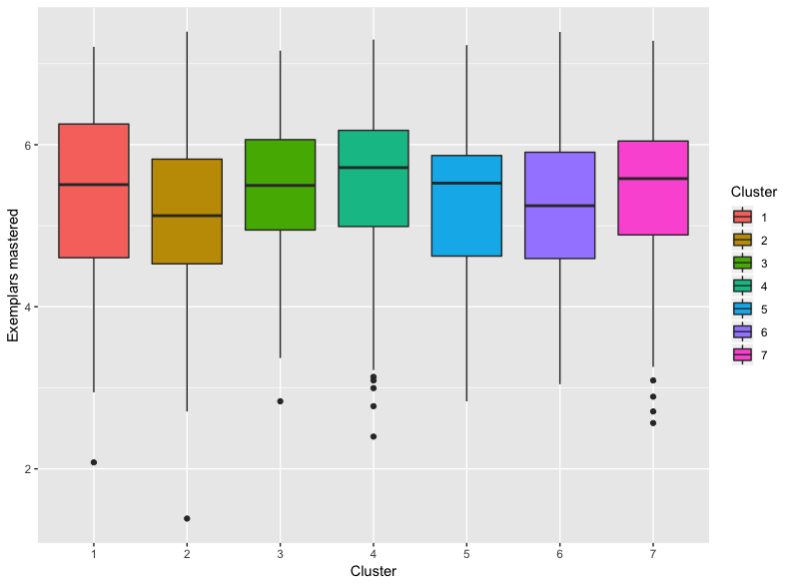
Box plots for each cluster. The box plots show the range of the exemplars mastered for each cluster, where the whiskers represent the minimum and maximum values. The line across each box is the median. The top of the box represents the third quartile. The bottom of the box represents the first quartile. Any points on the graph represent outliers in the clusters.

Box plots (Figure 5) for each of the 7 clusters depicts differences across clusters with respect to exemplar mastery and show the range of the exemplars mastered for each cluster, where the whiskers represent the minimum and maximum values (or 1.5 × the interquartile range, if outliers were present).

Increased treatment hours were associated with a significant increase in mastery (*P*<.001), and there were significant differences in mastery between clusters (*P*=.002); however, the interaction between treatment hours and cluster assignment was not significant (*P*=.28). Gender was nonsignificant (*P*=.051); however, the interaction between gender and cluster assignment did have a significant relationship with exemplar mastery (*P*=.018) (Table 3).

**Table 3:** Explanatory variables.

Variable	*P* value
Therapy hours	<.001
Gender	.051
Cluster	.002
Therapy hours × gender	.67
Therapy hours × cluster	.28
Gender and cluster	.02
Therapy hours, gender, and cluster	.63

Table 4 shows the averages for treatment hours and exemplars mastered for male, female, and combined clusters. Cluster 4 had the highest and Cluster 3 had the second-highest average number of exemplars mastered. Cluster 2 had the lowest average number of exemplars mastered.

**Table 4:** Average treatment hours and exemplars mastered across male, female, and combined gender clusters.

Cluster and dominant challenging behavior		Treatment hours	Exemplars mastered
**Male**			
	Tantrums	6.61	5.33
	Self-injurious behavior	6.64	5.05
	Elopement	6.71	5.41
	Stereotypy (low rate)	6.92	5.50
	Noncompliance	6.47	5.26
	Aggression	6.54	5.27
	Stereotypy (high rate)	6.72	5.41
**Female**			
	Tantrums	6.49	5.37
	Self-injurious behavior	6.72	5.43
	Elopement	6.66	5.50
	Stereotypy (low rate)	6.90	5.80
	Noncompliance	6.57	5.39
	Aggression	6.58	5.15
	Stereotypy (high rate)	6.66	5.13
**Combined**			
	Tantrums	6.58	5.34
	Self-injurious behavior	6.65	5.08
	Elopement	6.70	5.43
	Stereotypy (low rate)	6.91	5.55
	Noncompliance	6.50	5.29
	Aggression	6.55	5.24
	Stereotypy (high rate)	6.71	5.38

### Tukey Posthoc

Significant differences were found between Cluster 4 (low frequency stereotypy and moderate frequencies of other challenging behaviors) and Cluster 2 (self-injurious behavior) (*P*=.003) and between Cluster 4 and Cluster 6 (aggression) (*P*=.047). Overall, Cluster 4 had the highest rate of mastery while Cluster 2 had the lowest (Table 4); there was a significant difference between the clusters.

**Figure 6:**
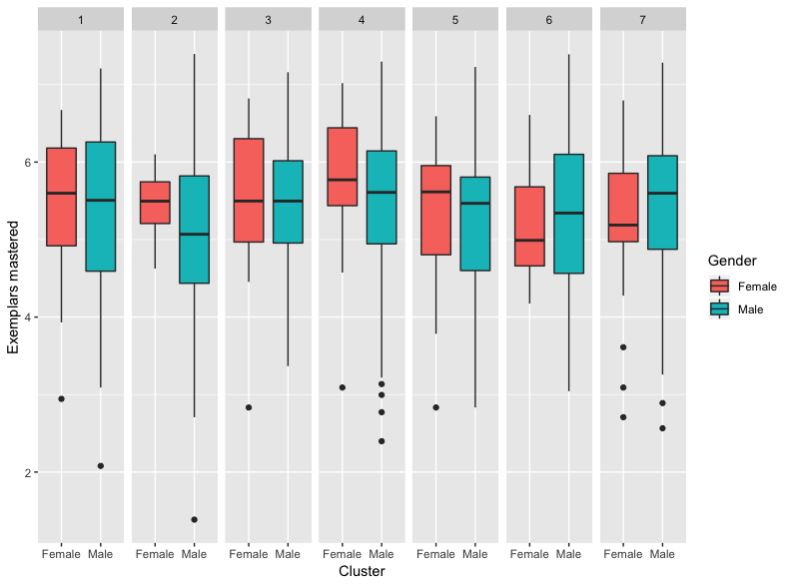
Box plots for each cluster and gender. The whiskers represent the minimum and maximum values. The line across each box is the median. The top of the box represents the third quartile. The bottom of the box represents the first quartile. Any points on the graph represent outliers in the clusters.

The interaction between gender and cluster assignment is depicted in Figure 6. Girls (*P*=.005) and boys (*P*=.03) in Cluster 4 mastered significantly more exemplars than the boys in Cluster 2. There was no significant difference between the girls in Cluster 2 and the girls and boys in Cluster 4. There were also no significant differences within clusters between genders (*P*=.003).

## Discussion

The purpose of this study was to identify types of autism spectrum disorder based on engagement in 8 challenging behaviors (ie, aggression, disruption, elopement, noncompliance, obsessive behavior, self-injurious behavior, stereotypy, and tantrums) as well as examine group and gender differences in treatment response; *k*-means clustering analyses performed on male, female, and blended samples revealed 7 unique clusters. These findings differ from those of Stevens and colleagues [56], in which 8 male and female clusters were identified based on engagement in challenging behaviors. Similar to those found by Stevens and colleagues [56], the clusters in our study were found to have a single dominant challenging behavior. Only 2 of the measured challenging behaviors (ie, disruption and obsessive behaviors) did not appear as a dominant challenging behavior across the identified clusters. Furthermore, relatively low rates of disruption and obsessive behaviors were also observed across all the clusters. Cluster 1 had tantrums as its dominant challenging behavior, Cluster 2 had self-injurious behavior as its dominant challenging behavior, Cluster 3 had elopement as its dominant challenging behavior, Cluster 4 had stereotypy (low rate compared to cluster 7) as its dominant challenging behavior, Cluster 5 had noncompliance as its dominant challenging behavior, Cluster 6 had aggression as its dominant challenging behavior, and Cluster 7 had stereotypy (at a higher rate than Cluster 4) as its dominant challenging behavior. Neither obsessive behavior nor disruption appeared as a dominant behavior in any of the clusters.

To explore the relationship between skill mastery, treatment hours, cluster assignment, and gender, multiple linear regression was performed. Interactions between all the explanatory variables were also evaluated. In line with previous findings [30,31], the relationship between skill acquisition and treatment hours was found to be significant in our study (*P*<.001).

In addition to treatment hours, cluster assignment was found to be significantly related to skill mastery (*P*=.002). Results from the Tukey posthoc test revealed that Cluster 4, characterized by the dominant behavior stereotypy with moderate frequencies of other challenging behaviors, was found to have significantly stronger levels of skill mastery than both Cluster 2, characterized by self-injurious behavior, and Cluster 6, characterized by aggression (*P*=.003). These findings suggest that treatment response varies across individuals with autism spectrum disorder that engage in different topographies of challenging behaviors. In particular, participants who engaged in self-injurious behavior and aggression were found to have poorer response to treatment compared to those with low levels of stereotypy. It is likely that prioritizing the treatment of self-injurious behavior and aggression using appropriate behavior interventions based on the identified function of the behavior [63] will result in better long-term treatment outcomes for these individuals.

The only interaction between explanatory variables that was found to be significant in this study was cluster assignment and gender (*P*=.018). No significant gender differences were found, with respect to skill acquisition, within the same cluster. That is, boys and girls in the same cluster were found to have similar rates of skill acquisition (Table 4). Significant gender differences were found across clusters, however. Specifically, both girls and boys in Cluster 4 (stereotypy) displayed stronger rates of skill mastery than boys in Cluster 2 (self-injurious behavior); however, no significant differences were found between boys and girls in Cluster 4 and girls in Cluster 2. In previous research, gender was found to be a risk factor for the occurrence of challenging behaviors in individuals with autism spectrum disorder [8-12]. While the role of gender is unclear, this finding provides further support for the significant differences in treatment response across clusters, particularly for Cluster 4 and Cluster 2.

This study has several limitations that are important to consider. As a retrospective study, the analysis was limited to the existing data in the data set. Data on race and ethnicity were not available in the data set; therefore, representation across those demographics and any potential disparities in this sample are unknown. Furthermore, variables such as autism spectrum disorder symptom severity and IQ were not measured. Both symptom severity and IQ have been found to be related to engagement in challenging behaviors [2,3,7-9] as well as related to treatment response [27,35,36,38-45]. In particular, aggression and self-injurious behavior, the behaviors associated with slower skill acquisition in our study, have been linked to low IQ scores [8]. It would be worth exploring these variables in future research. In addition, the method used to aggregate the data for clustering results in a relatively small feature space of only 8 dimensions. These dimensions correspond to broad categories of challenging behaviors but do not capture other aspects related to those behaviors such as function. A future study could improve on this work by starting with a higher-dimension behavioral feature space, including functions of behavior, and then utilizing contemporary feature selection algorithms to derive the most meaningful subset of features to be fed to the unsupervised learning algorithm. In this case, use of a clustering algorithm that is more sophisticated may be warranted; the *k*-means algorithm takes a simple approach to clustering that relies upon regularly shaped clusters throughout the feature space. Finally, we note that additional studies to validate the clusters identified here would be valuable. In particular, the use of an additional cohort of participants which could be assigned to clusters and then have their assignments verified by clinicians using a broader set of medical records would be important to verify that the clusters identified here are generalizable beyond the study population.

This study is among the first to investigate types of autism spectrum disorder based on engagement in challenging behaviors and the impact of challenging behaviors on treatment response. Findings suggest that challenging behaviors do impact treatment response with specific topographies (ie, self-injurious behavior, aggression) being particularly detrimental. In future investigations, it would be worthwhile to map the function of the behavior (eg, attention, escape, tangible, automatic), in addition to the topography, and explore its impact on treatment response. Future research should also explore targeted interventions to improve skill acquisition based on cluster assignment, particularly for the clusters characterized by self-injurious behavior and aggression. Until such investigations are conducted, treatment providers should be aware that these behaviors seem to have a particularly negative impact on skill acquisition and interventions addressing these behaviors may be worth prioritizing in treatment. To further improve outcomes across individuals with autism spectrum disorder, attention must be given to segmentation within the disorder. Investigations, such as these, show the promise of unsupervised machine learning models in identifying types of autism spectrum disorder so that targeted treatments based on type membership may be explored. We recommend that clinicians who are interested in further exploring latent structural features of the autism spectrum, including challenging behaviors, proactively collect data to the greatest extent that is practical and unobtrusive. Such data, especially in aggregate, will be essential for gaining additional insights into autism spectrum disorder types with the ultimate goal of personalizing and optimizing treatment plans.
